# Analysis of Incremental Sheet Forming of Aluminum Alloy

**DOI:** 10.3390/ma16196371

**Published:** 2023-09-23

**Authors:** Costel Catalin Coman, Simona-Nicoleta Mazurchevici, Constantin Carausu, Dumitru Nedelcu

**Affiliations:** Faculty of Machine Manufacturing and Industrial Management, Machine Manufacturing Technology Department, “Gheorghe Asachi” Technical University of Iasi, Blvd. Mangeron 59A, 700050 Iasi, Romania; catalin.coman06@gmail.com (C.C.C.); c_carausu@yahoo.com (C.C.); nedelcu1967@yahoo.com (D.N.)

**Keywords:** incremental forming, sheet, Al 3003, ANOVA analysis, deformation forces

## Abstract

Recent developments in incremental sheet forming have resulted in the creation of novel manufacturing processes that are highly adaptable and could bring significant economic benefits for advanced technologies and low-volume production. In this manuscript, the following variables were examined: the variation in the deformation forces for a part with a pyramidal trunk shape; the variation in the deformations and thinning of the Al 3003 material during the incremental forming process; and the variation in the accuracy of the incrementally formed part and the quality of the surfaces (surface roughness). The components of the forces in the incremental forming have increasing values from the beginning of the process to the maximum value due to the hardening process. The TiN-coated tool ensures lower values of the forming components. Due to the kinematics of the forming process, deviations, especially in shape, from the part in the drawing are observed, which are shown by the radius of curvature of the side wall of the part, the appearance of a radius of connection between the wall and the bottom of the part, as well as dimensional deviations that are expressed by the variation in the forming depth. Concerning the smoothness of the surfaces, it was observed that the best roughness results were obtained in the case of the TiN-coated tool.

## 1. Introduction

Incremental sheet forming (ISF) is a novel and adaptable technology for prototyping and small-lot production.

A classification of the ISF process is conventional and hybrid methods with a brief description as follows:

- Single-point incremental forming—one shaping or deforming tool passes on a sheet through a series of tiny progressive deformations until the desired shape is obtained [1,2,3,4,5,6];
- Two-point incremental forming—two forming tools are used: one deforms the sheet into the appropriate geometry, while the other one supports the sheet from the rear [7,8,9];
- Multi-point incremental forming (flexible 3D manufacturing process)—used for big sheets and has two opposed hard dice (lower and upper) with systems consisting of multiple punches with the highly precise movements that are necessary to obtain the desired shape being carried out with linear actuators [10,11,12];
- Single-point incremental forming with hydraulic fluid—the forming tool is on one side, and the hydraulic fluid pumped to a high pressure is on the opposite side [13,14,15];
- Two-point incremental forming with partial die—the sheet is formed using a partial die on one side and a moving forming tool on the other [16,17,18];
- Two-point incremental forming with full die—a complete die is applied to one side of the sheet, while the forming tool rotates on the other side [19,20,21,22].

The process parameters involved in ISF encompass various factors, such as tool path, wall angle and tilting angle, incrementation/step depth, speed of rotation, tool diameter, temperature, and lubrication. These parameters interact with each other, influencing the overall outcome of the ISF process. Other key output parameters are also taken into consideration, such as the following [23,24,25,26]:

- forming force—There are three types of forces that contribute to sheet deformation: those derived through prediction, analysis, and experimentation. Finally, the forming force shifts when different inputs are used [27];
- formability—The evaluation of a material’s deformation characteristics can be conducted using various methods, such as the swift cup drawing test and the Erichsen cupping test [27,28];
- surface roughness—The tool radius, the initial roughness of the active part of the tool, the thickness of the sheet, the flow rate, the incremental depth, and the various tool designs are all process characteristics that might affect surface roughness [29];
- spring back—There are two types of spring back: local and global. The first one produces poor accuracy, and the second produces residual stresses [30];
- failure to fracture—During the process of sheet material deformation, it undergoes multiple stages that ultimately lead to the fracture stage [31].

The vast majority of incremental sheet-forming applications have been carried out in the metalworking industry, and the most significant raw materials that are used currently in the industry are mild steel (small-batch production or rapid prototyping); aluminum/aluminium alloys (frequently used due to small forming loads); stainless steel/high-strength steels (limited by difficulties related to significant elastic spring back); copper, brass, and titanium (heat up during forming—blister appears); magnesium; platinum alloys; polymers (not really suitable—short life cycles but, by using single-point incremental forming, this raw material can be considered); and even sandwich panels (sheets from different materials as steel/polymer/steel or aluminum/polymer/aluminum with a lightweight core that improves buoyancy, impact absorption, thermal insulation, vibration absorption, and sound-deadening) [32,33,34,35,36,37,38,39].

Single-point incremental forming is an improvement over traditional sheet forming because it does not require the use of dies. For low-volume production items, the reduction in die can save tooling costs while increasing turnaround time. Die for the two-point incremental forming procedure can be constructed from cheap materials like wood, allowing for a low total tooling cost. However, this method is inherently time-consuming, and it often results in diminished dimensional accuracy—especially for intricate components. When it comes to the construction of automobile prototypes and the manufacture of parts in small quantities, incremental sheet forming is a method that is both practical and cost-effective [40,41,42,43].

The researchers are conducting different studies in the ISF area in order to explain some behaviors or to observe, for instance, the influence of process parameters on the obtained part characteristics.

The mechanism of sheet deformation is associated with the shearing and stretching that appear in the plane perpendicular to the direction of the tool and the shear parallel to it [1,44]; also, other researchers [25] consider that this is produced with a mixture of spinning and stretching.

The reduction in part size to the micro domain is another goal, achieved by using devices installed on a scanning electron microscope [45], a conventional CNC machine modified to function as a micro incremental sheet forming system [46], different types of thin sheets from classic alloys (aluminum, cooper), stainless steels, and silver or titanium [47,48,49,50,51].

The physical and mechanical characteristics of the sheet are significantly influenced by the microscale and macroscale forming processes [52,53]. Also, classic determination of mechanical properties was realized on deformed sheets: tensile test at different temperatures [54,55], yield strength, ultimate tensile strength, total elongation [56,57], spring back defect [58], the phenomenon of shape distortion resulting from supplementary heat treatment [59], and the effect of lubrication [60,61].

From a simulation point of view, the studies are focused on the sequential limit model for thickness distribution [62], influence of the thickness and average grain size ratio on the yield stress and tangent modulus [63], finite element model (FEM) to observe deformations and elasto-plastic behavior material [64], FEM that simulate the impact of plastic deformation on the force precision [40], and three-dimensional FEM that incorporates both elastic and plastic material behavior for three completely different materials (wood, polymer, and rubber) [65,66,67].

In order to highlight even more, the studies carried out so far on single-incremental forming (SPIF) have been selected from the specialized literature (Table 1).

The main problems that are not solved by the previous researchers are the particularities of incremental forming in a single point for obtaining parts in the pyramid trunk form (without revolution movements) and incremental forming with titanium nitride-coated tools in order to observe their characteristics.

No research has been carried out on forming processing performed with tools coated with titanium nitride led by industrial robots. This type of processing is different from that performed with CNC milling machines due to the lack of rotation movement of the tool as well as a lower rigidity of the technological system.

The original contribution of this study is that tools coated with TiN (used in the forming process) allow for an improvement of the results of the incremental forming process of a part with a pyramid trunk shape.

Extensive experimental investigations and numerical simulations have been conducted to study the incremental sheet forming (ISF) process. These studies have contributed to establishing a comprehensive understanding of the deformation mechanism involved in macro ISF. In this manuscript, we analyzed the variation in the deformation forces for a part with a pyramid trunk shape; performed ANOVA analysis of the experimental results regarding the maximum components of force in the incremental forming process; and analyzed the variation in the accuracy of the incrementally forming part and the quality of the obtained surfaces (surface roughness).

## 2. Materials and Methods

### 2.1. Materials

The semi-finished products used to obtain the parts with incremental stamping were constructed of sheet materials of EN AW 3003 aluminium alloys with a thickness of 1.8 mm. The main mechanical and physical properties of EN AW 3003 aluminium alloy (AlMnCu DIN 3.0517) used for incremental drawing are the following [94]: tensile strength (120–160) MPa; yield strength—min 85 MPa; elongation at break (sheet thickness 1.2–6.3 mm)—6% (out of 50 mm); hardness—36 HB; density—2.73 (g/cm^3^); modulus of elasticity—69 GPa; thermal conductivity at 25 °C—193W/m.K; melting point—655 °C; and coefficient of thermal expansion (20–100 °C)—23.2 μm/m/°C. The geometric features of the sheet were the following: thickness—1.8 mm; length and width before cutting (6000 × 2000) mm; length and width after cutting (240 × 200) mm; forming depth—200 mm; large base of the pyramid trunk—a square with 85 mm side; and pyramid wall inclination angle—55°.

The material used for the execution of the incremental forming tool was X153CrMoV12 steel with the following mechanical and physical properties [95]: tensile strength—860 MPa; yield strength—420 MPa; density 7.7 (g/cm^3^); modulus of elasticity—210 GPa; thermal conductivity at 350 °C—21 W/m∙K; coefficient of thermal expansion (at 20 °C)—13 μm/m/°C; and hardness—54–60 HRC.

**Figure 1:**
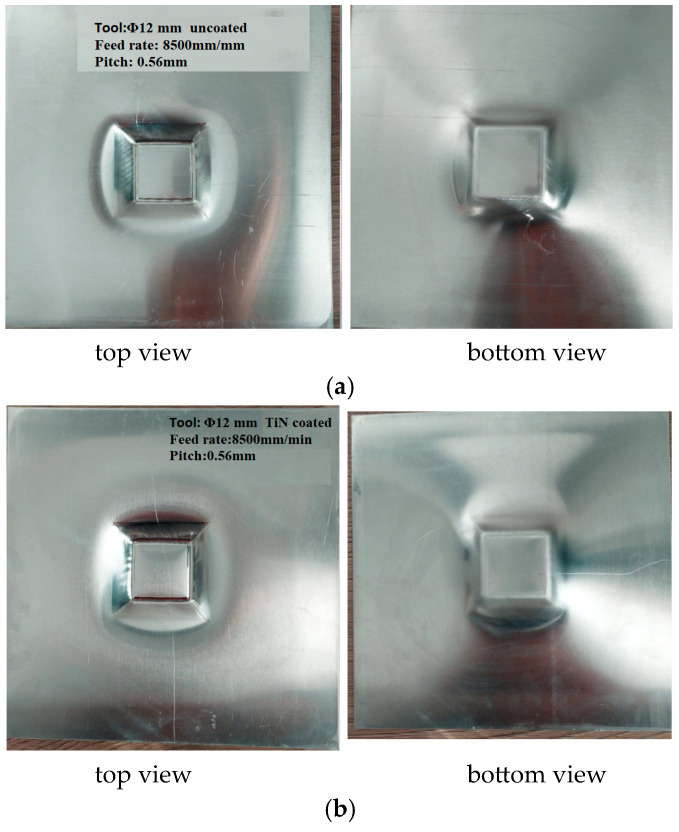
Images of the semi-finished products after processing. (**a**) uncoated tool. (**b**) TiN-coated tool.

Following the processing with spirit, the produced parts are presented in Figure 1.

### 2.2. Machine Tools, Equipment, and Tools

The machines, equipment, and tools used in the incremental ambush were as follows:

✓ Kuka KR210-2 6-axis robot, developing a maximum load of 2100 N with a positioning repeatability of ±0.06 mm. It is equipped with a KR C2 controller (KUKA Roboter GmbH, Augsburg, Germany), and the numerical programming software is SprutCam (X version, SprutCAM Tech, Limassol, Cyprus) [96,97]. This robot, together with the Aramis measurement system, was used for measuring deformations and wall thinning;
✓ Incremental forming force measurement device. A measuring system, consisting of the PCB261A13 sensor (PCB Piezotronics, Inc., NY, USA), the CMD600 signal amplifier (KUKA Roboter GmbH, Augsburg, Germany), and the Quantum X MX840B acquisition system (Hottinger Brüel & Kjær, Naerum, Denmark), was used to measure the forces during the incremental forming process. The force sensor makes it possible to acquire forces in dynamic and quasi-static format, and the amplifier creates the signal voltages required for acquisition with the Quantum X system. The force transducer consists of a piezoelectric sensor assembled between 2 plates.

The CMD600 amplifier was used to obtain ±10 V voltages on the signal acquired from the force sensor PCB261A13 [98]. Three such amplifiers were used for the experimental research, one on each channel (for each of the forces F_z_, F_x_, and F_y_).

The Quantum X MX840B acquisition system [99] has 8 individually configurable and electrically isolated measurement channels. For each channel, 16 types of transducers can be connected.

The OGP SMARTSCOPE 600 FLASH (OGP Messtechnik GmbH, Wallau, Germany) and Formtracer Avant FTA-H8 D4000-D (Mitutoyo, Sakado, Japan) measuring machine were used for the measurement of the acquired parts.

The power tools used for the preparation of the incremental forming process were the following: angle grinder Panzer AP125-12 (Panzer, Germany); and the drilling and screwing machine MAKITA DF347DWE (Makita Corporation, Anjo, Japan);

A 10W40 engine oil (MOTUL, Aubervilliers, France) was chosen as the lubricant for better stretching of the material during the incremental forming process.

The tools used were of two types as follows: simple ball-tip tool (uncoated tool) and a ball-tip tool coated with TiN titanium nitride (coated tool). In the case of both tools, the ball had a diameter of Ø12 mm and a hardness of (53–58) HRC. The TiN coating has the advantage of good wear and corrosion resistance. The hard material applied without a metallic binder has a high hardness, i.e., it has an extremely fine grain size that allows for very good friction resistance [100]. The hardness of the TiN layer is approximately 2300 HV, and the coating thickness is (2–4) μm.

**Figure 2:**
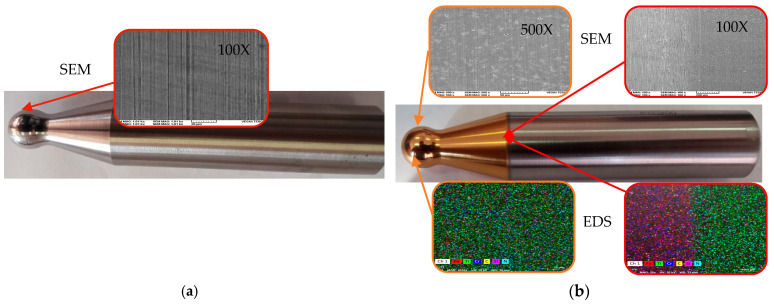
Tools used for SPIF. (**a**) uncoated tool. (**b**) TiN-coated tool.

To determine the microstructure of the tools, SEM and EDS analyses were performed using VegaTescan LMHII equipment (TESCAN ORSAY HOLDING, Kohoutovice, Czech Republic) with an EDX detector X Flash 6I10 from Bruker, Germany, Esprit 2.2 software. Figure 2 shows the two tools, the uncoated and the coated one. The SEM image of the uncoated tool (after use) highlights visible traces of wear. The sample coated with titanium nitride, according to the SEM and EDS analyses, reveals a uniform deposition of the layer but also an insensible wear of the tool after use.

The Solidworks 2018 software package was used for the 3D modeling of the parts as well as for the execution drawings.

In order to perform the finite element modelling, the software package Abaqus/CAE version 6.14 was used.

### 2.3. Methods

The Erichsen method was used for tensile/compression testing of the semi-finished product. The specimens used for the punching test were rectangular with one side 50 mm and the other side 300 mm. The condition of the test sample is prescribed by the respective material standards. No machining of the samples was carried out prior to the test because machining may affect the material’s ability to be drawn and influence the test results. Sample deformation in the Erichsen equipment was performed with radial and circumferential stretching [101].

The experiments were conducted following a full factorial experimental design with three influencing factors, each on 2 levels, of type 2^3^ = 8 experiments. The factors considered were the tool (−1—uncoated tool; +1—TiN-coated tool), feed rate (−1—5500 mm/min; +1—8500 mm/min), and increment step (−1—0.28 mm; +1—0.56 mm).

Table 2 shows the experimental results of the maxima of the force components in the full factorial design.

**Figure 3:**
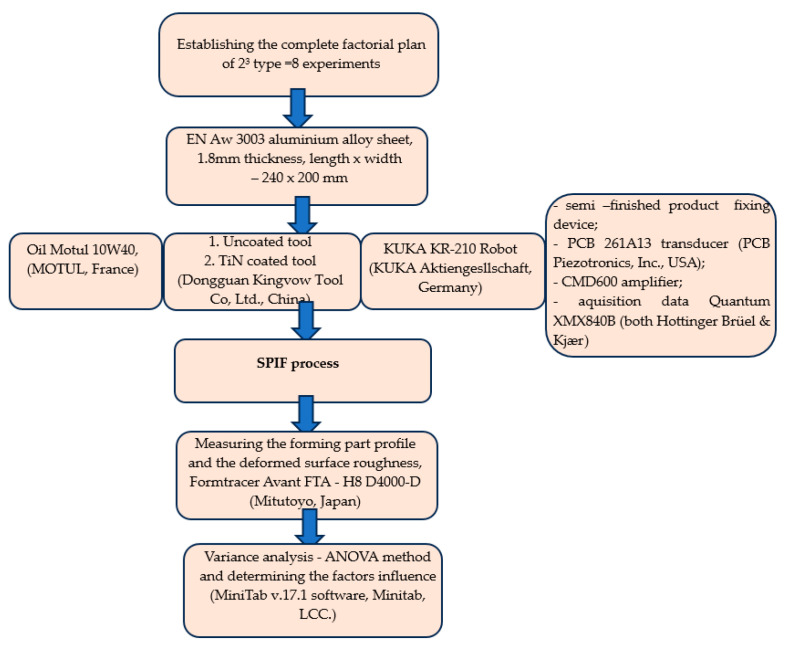
Flow chart of the experimental procedure.

The flowchart of the conducted experiments is presented synthetically in Figure 3.

The ANOVA method with the MiniTab software (17.1.0 version, Minitab, LLC, State College, PA, USA) package was used for statistical processing and determining the influence of factors, and the LS-DYNA software package (3.2 version, Livermore Software Technology Corporation (LSTC), Livermore, CA, USA) was used for numerical simulation.

The type of problem required for the selected simulation from the LS-DYNA program was Forming. The created model was composed of the following elements: semi-finished product, active plate, and withholding plate and tool—modeled in the form of a sphere.

**Figure 4:**
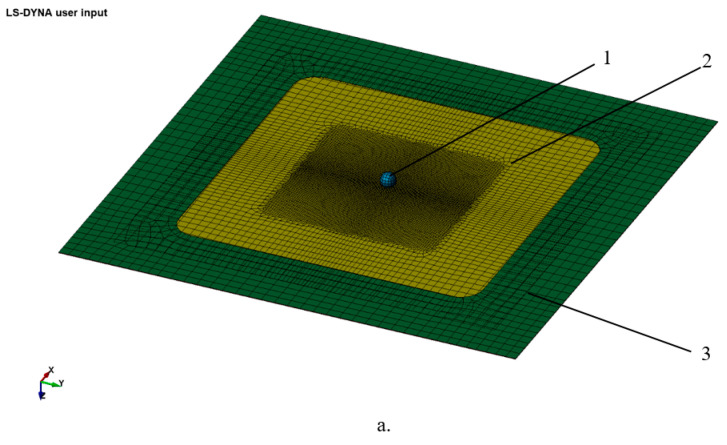

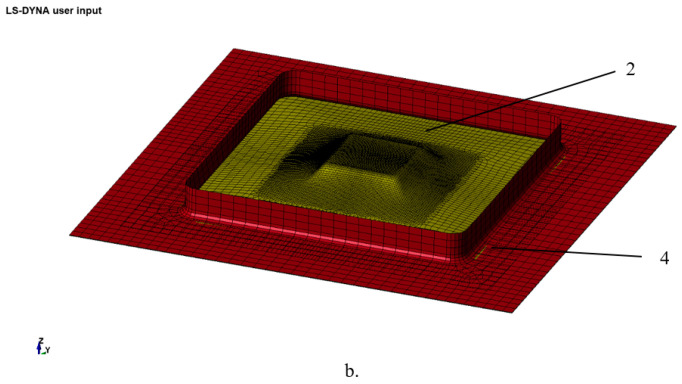
Simulation model: (**a**) at the beginning of the incremental forming process; (**b**) at the end of the process; 1—tool; 2—semi-finished product; 3—restraint ring; 4—active plate.

Figure 4 shows the model used to define the problem in two situations: at the beginning of the incremental forming process (Figure 4a) and at the end of the processing (Figure 4b).

The semi-finished product was discreet with elements of Shell type (with the help of the Elform parameter) for which the explicit formulation #16 was chosen, forms that ensure the calculation of the deformation forces but also an increase in the solving time. The number of integration points by thickness, nip, was chosen as 7, which allowed for a high accuracy to the detriment of increasing the running time.

For the other two boards and for the sphere, the same type of element (Shell) was chosen but with two integration points.

For discretization, the method of adaptive refinement was used, starting from a network of coarse finished elements. At each iterative step, each element from the previous step was divided into four smaller finished elements.

Three contacts of the type “forming-other-wayfaceosurface” were generated between the electric elements: between the sphere (tool) and the semi-finished product, between the active and semi-finished plate, respectively, and between the withholding and semi-finished plate. In order to solve the contact problem, the penalty method was applied.

The kinematics of the tool was defined with the key word *Boundery_Prescriebed_Motion_Rigid, which allowed for the direction of movement and speed by specifying the movement curves after the three axes. The action of the retention plate was modeled by applying a 2000 N press force in the Z direction.

The rubbing between the tool and the semi-finished product was modeled by adopting a friction coefficient, which allowed for the differentiation of the use of the two types of tools. Thus, for the uncoated tool, the coefficient of friction was 0.09, while for the coated tool, the coefficient was 0.075. The running time on the computer that was required to solve the problem (either with an uncoated or coated tool) was 68 h.

## 3. Results

### 3.1. Numerical Simulation of the Variation in the Components of the Ambulation Force

**Figure 5:**
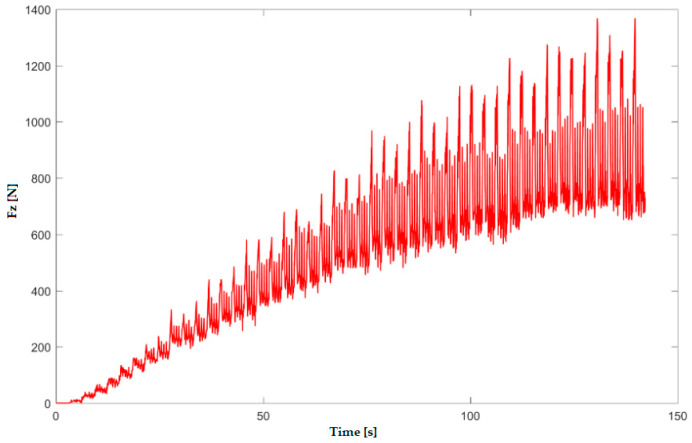
Variation in F_z_ force for case 1—uncoated tool—obtained with numerical simulation with LS-Dyna.

**Figure 6:**
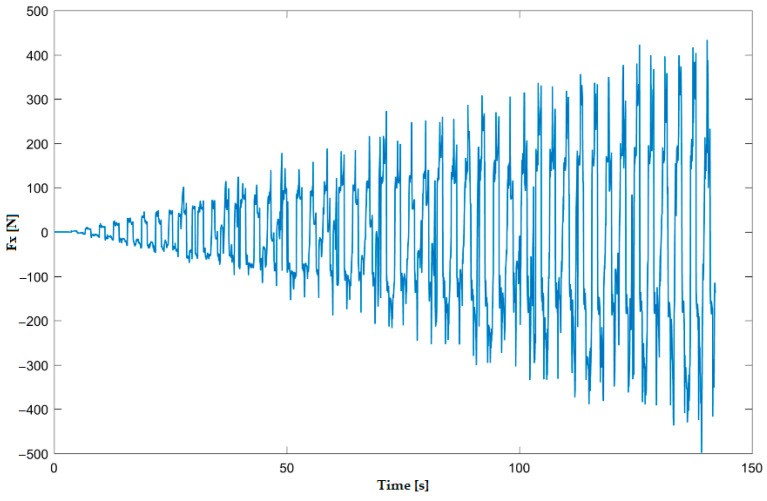
Variation in F_x_ force for case 1—uncoated tool—obtained with numerical simulation with LS-Dyna.

**Figure 7:**
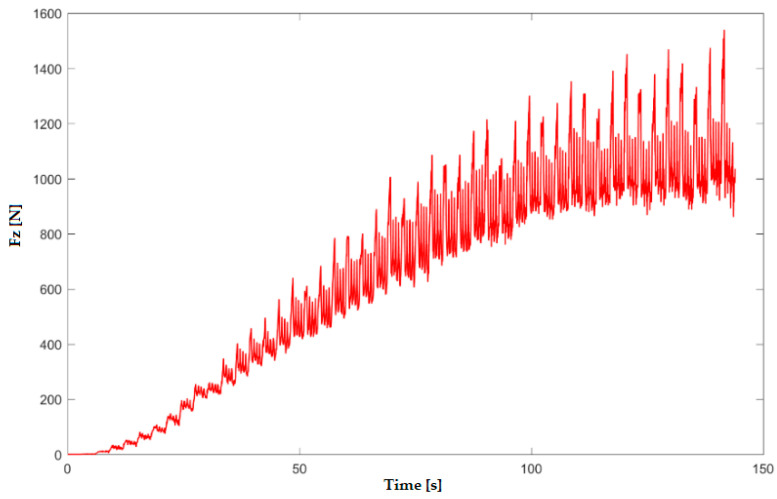
Variation in F_z_ force for case 2—coated tool with TIN—obtained with numerical simulation with LS-Dyna.

**Figure 8:**
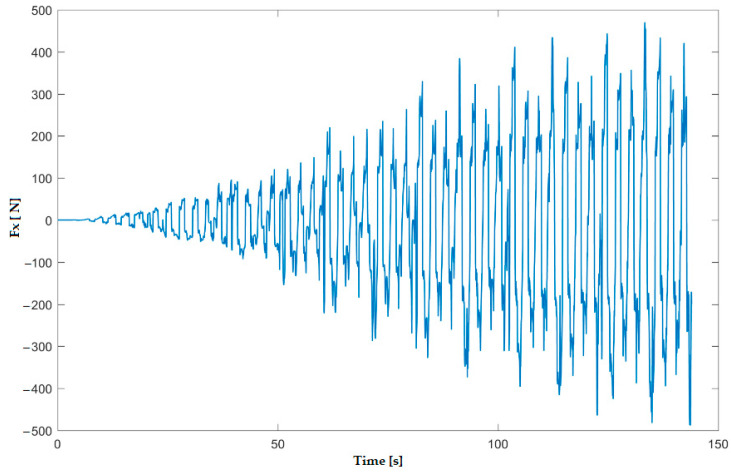
Variation in F_x_ force for case 2—coated tool with TiN— obtained with numerical simulation with LS-Dyna.

In the numerical simulation, two cases were considered as follows: case 1 corresponds to the uncoated tool (Figure 5 and Figure 6), and case 2 corresponds to the tool coated with TIN (Figure 7 and Figure 8). Thus, the F_z_ component (Figure 1 and Figure 3) increases from zero towards the maximum value, reaching the maximum towards the end of the working stroke but also having a series of local maxima at each change in direction of the plane of the tool. The F_x_ component (Figure 2 and Figure 4) and F_y_ have the same type of variation, lagging each other, going from negative to positive values depending on the direction the tool is moving at a given time. For both the F_x_ and F_y_ components, the maximum value is obtained at the end of the tool’s working tool.

### 3.2. Influence of Working Parameters on the Components of the Forming Force

The deformation force is an important process parameter during the incremental forming process, which has a major influence on the machining results of the parts. The deformation forces in incremental forming are relatively small compared to those in classical forming but are an important factor when using CNC machining centers or industrial robots. The forming forces used in the incremental forming process depend largely on the depth of forming, the tool diameter, the angle of the part being formed, the blank used and its thickness, and the lubricants used; the axial force F_z_ is not influenced by the rotational speed of the tool and the lubrication of the blank during the forming process—only by the radial force F_x_ [64].

For comparison with the results obtained from the numerical simulation, the experimental results of experiments 2 and 6 in the experimental design will be presented.

**Figure 9:**
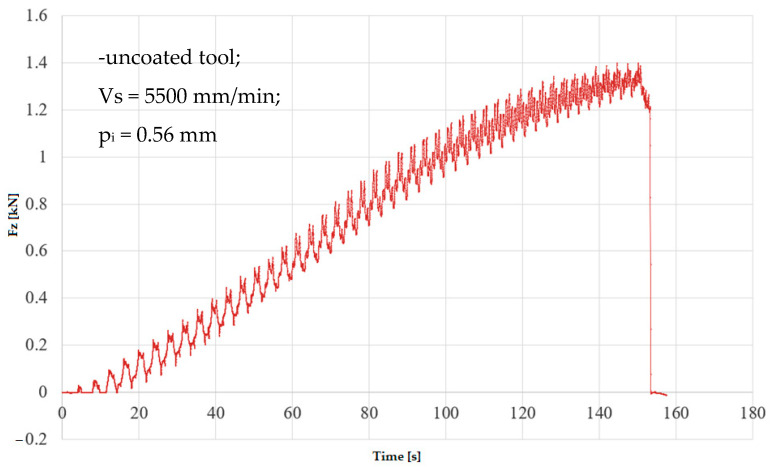
Variation in F_z_ force in experiment 2.

**Figure 10:**
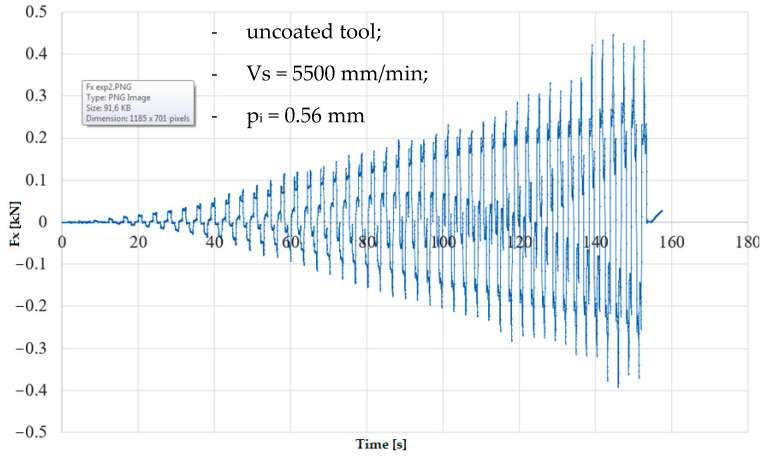
Variation in F_x_ force in experiment 2.

In Figure 9 and Figure 10, the variation in the components F_z_ and F_x_ along the direction of the two coordinate axes XZ in experiment 2 is shown graphically. The forming time was 157.4 s; this was lower because the incremental step was double, and the number of passes was lower. The maximum forming force F_z_ obtained in experiment 2 is 1.39 kN; the maximum value obtained for the F_x_ force is 0.38 kN.

**Figure 11:**
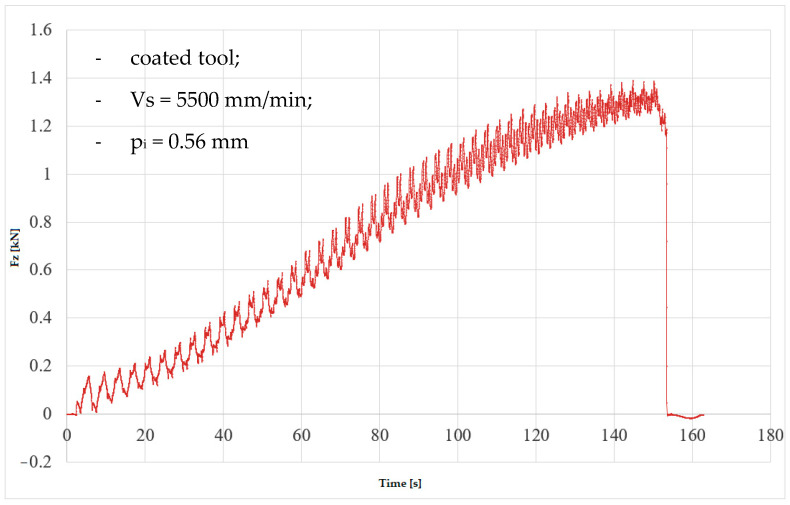
Variation in F_z_ force in experiment 6.

**Figure 12:**
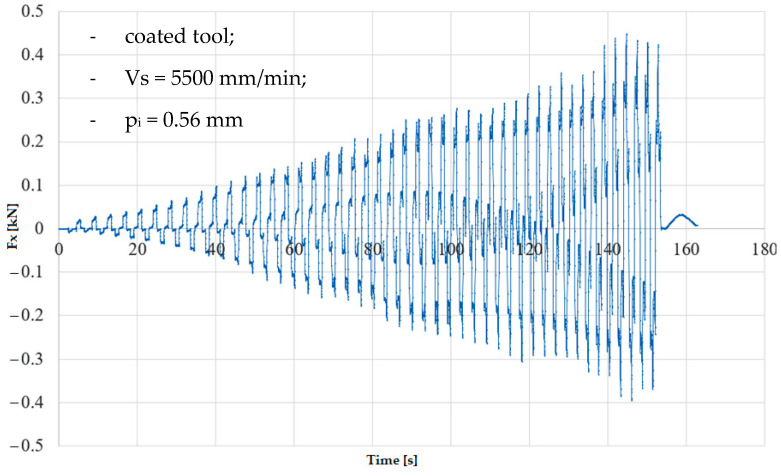
Variation in F_x_ force in experiment 6.

In Figure 11 and Figure 12, the variation in the force components F_z_ and F_x_ along the direction of the three XYZ coordinate axes for experiment 6 (case 2 of the coated tool) is plotted. Thus, the maximum values of the forces are as follows: F_z_ reaches the maximum value of 1.38 kN, and F_x_ reaches the maximum value of 0.42 kN.

After running the numerical analyses with the finite element method, it was possible to compare the results obtained theoretically (numerically) with those obtained experimentally. A summary of these results and, especially, of the differences obtained between the numerical and experimental results is presented in Table 3 (comparative results for the uncoated tool, case 1 of the simulation and experiment 2) and Table 4 (comparative results for the TIN-coated tool, case 2 of the simulation and experiment 6).

It should be mentioned at the outset that the results obtained for the force components in the numerical simulations have a higher percentage than those obtained for the deformations due to the fact that the program calculates these components as being those of the reaction occurring between the nodes of the tool and those of the blank. However, as can be observed from Table 3 and Table 4, the differences are acceptable with the highest values being 7.97% and 7.89%, respectively. From the analysis of the data presented in Table 3 and Table 4, it can be observed that the values for case 2 are higher in the OX and OY directions and lower for the force in the OZ direction than those for the forces obtained in case 1. In conclusion, it can be stated that the simulation using the finite element method leads to results with a relatively low percentage of error and can be successfully used for numerical simulation of the incremental buckling process.

**Figure 13:**
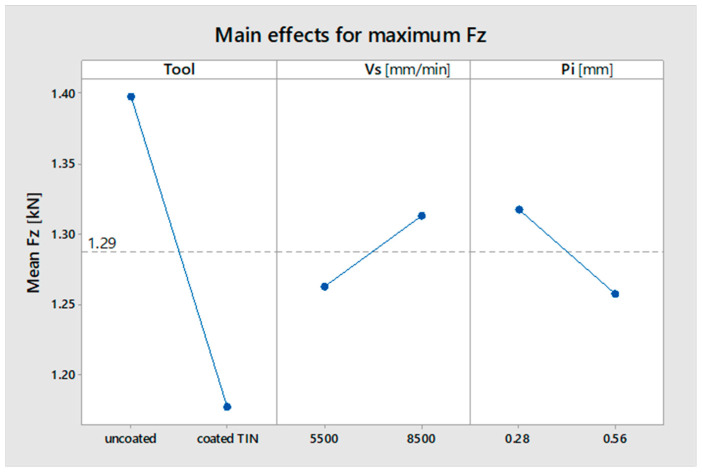
Influence of factors on the maximum force component F_z_.

The influence on the maximum force component F_z_ is shown in Table 5, and the influence graph is shown in Figure 13.

Tool is the only factor that has a statistically significant influence on the magnitude of the F_z_ force (significance threshold *p* < 0.026). The other factors have no statistically significant influence (*p* > 0.03).

The TIN-coated tool provides a lower maximum force F_z_ during incremental forming than when using the uncoated tool.

**Figure 14:**
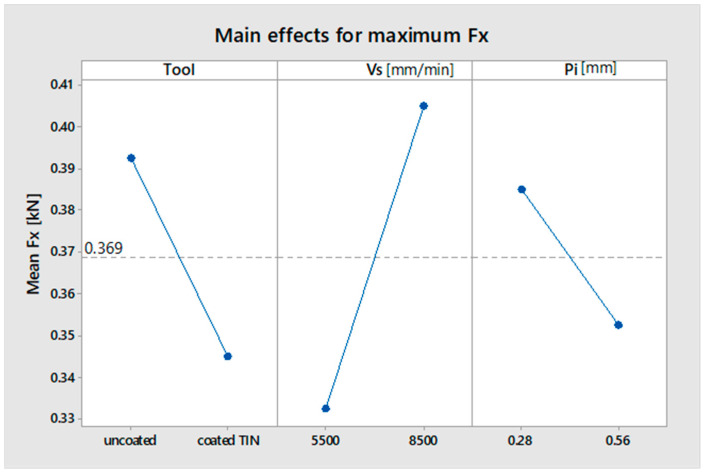
Influence of factors on the maximum force component F_x_.

The influence on the maximum force F_x_ is shown in Table 6, and the influence graph is shown in Figure 14. According to Table 6, none of the factors has a statistically significant influence.

It is found that the value of the maximum force component F_x_ increases with increasing forward speed.

As in the case of the maximum value of the F_x_ force, a similar influence of the analyzed factors is found.

Due to the large variation in the disturbances influence in the case of maximum values of the forces components (obtained at the end of the forming process), the experimental results have variations that have a high dispersion, which determines that the advance speed influences and the incremental step are not statistically insignificant in the case of the F_z_ component. In the case of the X and Y directions (the tool movement directions in the perpendicular plane to the forming direction), no parameter is statistically significant, and the variability of these components is even greater. One solution would be to increase the distance between the two levels (−1 and +1) of these parameters but only for quantitative parameters (speed advance and increment). Thus, in the study carried out by [102] with the same type of material as in the present paper, by the statistical processing of the experimental results with the MiniTab software 17.1.0 version, the increment step influence on the F_z_ component came out strongly significant (*p* = 0.01). The result was obtained due to the fact that the difference between the two levels was seven times (0.1 mm and 0.7 mm, respectively).

Disturbance can also occur due to the variability of the parameters of the technological system.

### 3.3. Execution Accuracy

**Figure 15:**
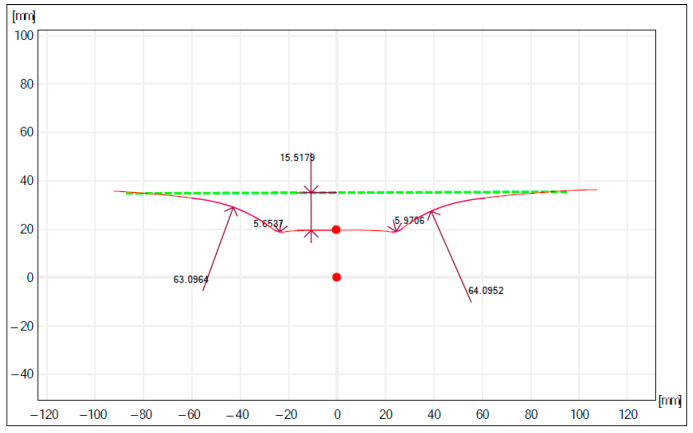
Measurement of the part profile obtained in experiment 2.

**Figure 16:**
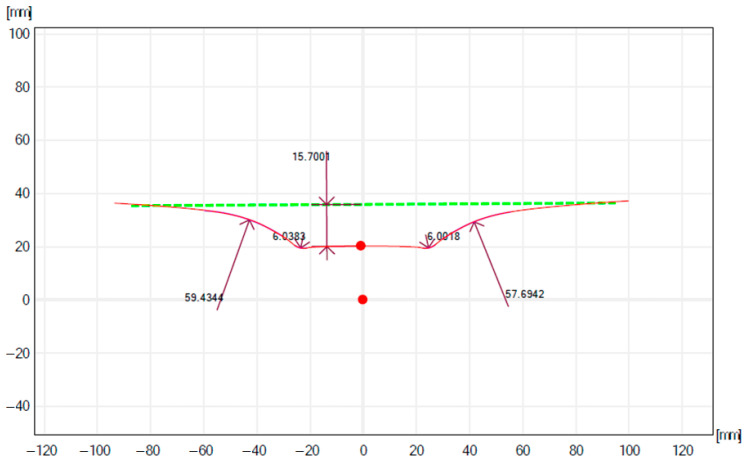
Measurement of the part profile obtained in experiment 6.

After the parts are produced with the incremental forming process, the measurement of the parts obtained is carried out. For the measurement of the depth of the part, the measuring equipment took, as a basis, a theoretical straight area as can be observed in Figure 15 and Figure 16.

In the study [103], the geometric accuracy of the pieces processed by using SPIF is analyzed, underlining that the geometric and form deviations are produced as a result of unwanted plastic deformations, spring back, and the pillow effect. The study does not signal the deviation from the rectilinearity of the wall of the part obtained with forming. The present study signals the appearance of a wall form deviation of convex shape of wall curvature. This form deviation occurs due to the forming force variation during the process, the increase in the cold hardening (due to the spring back reduction), and the material thinning; thus, at the beginning of processing, the F_z_ is smaller, and the deformation of the material is lower, which leads to a smaller forming angle [104]. At the maximum forming depth, the wall thinning is maximum, and the forming force is maximum, which determines a higher deformation degree, thus, obtaining a maximum forming angle.

The deviation from the forming depth is due to spring back and has relatively high values due to the SPIF (one tool). The reduction solutions are the use of the counter-tool and the introduction in the deformation area of a controlled temperature of 200–300 °C.

Another cause of deviations is the use of the robot (compared to milling CNC) that, according to [105], induces position errors of the tool center that are caused by the elastic deformations to which the robot is subjected to during the process.

### 3.4. Quality of the Surfaces Obtained

**Figure 17:**
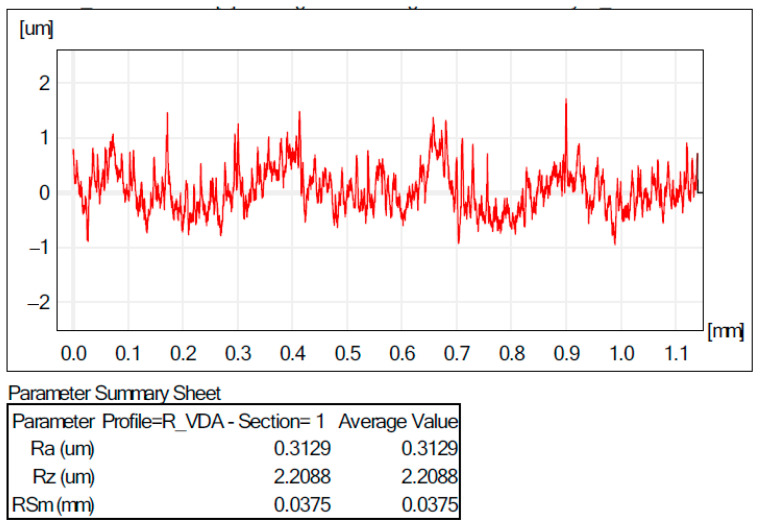
Roughness values for the part in experiment 2.

**Figure 18:**
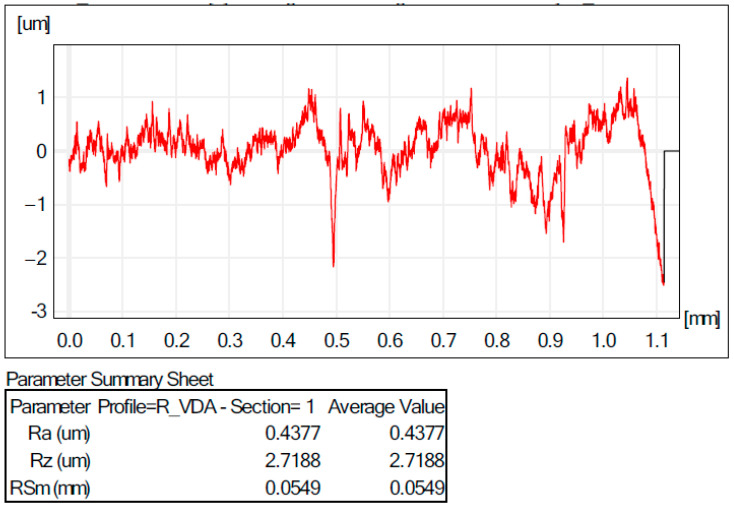
Roughness values for the part in experiment 6.

The quality of the surfaces obtained with the incremental forming process is influenced by a number of factors, such as the surface quality of the blank and the die and their shape deviations. Another key factor is the process parameters used in the incremental forming process. If all these factors have been checked, the next step is to determine how the process is to be carried out with or without lubrication, as friction between the tool and the blank causes a temperature rise; for this reason, we can have significant influences on the roughness of the machined surface. A very important thing is the choice of the type of lubricant that meets all the conditions to allow the tool to stretch the material so that there will be influence on the quality of the incrementally ground surface. In this work, an aluminium alloy blank was used, where it was taken into account to avoid, as much as possible, material deposits on the tool surface by choosing 10W40 engine oil, which could have a significant influence on the roughness of the incrementally ground surface. In order to check the quality of the surface obtained, the roughness R_a_ (μm) and R_z_ (μm) were measured (Figure 17 and Figure 18) for all the parts obtained experimentally. It can be observed that, in the case of the TiN-coated tool, a better smoothness of the surfaces of the obtained parts was obtained.

An improved smoothness of the surfaces was attained in the tool case with the TiN coating due to a superior superficial hardness that reduced the local deformation in the contact area, tool semi-finished product, and, as a consequence, the contact area. On the other hand, the initial roughness of the tool coated with TiN was lower (R_a_ = 0.2 μm) compared to that of the uncoated tool that had the parameter of the roughness R_a_ = 0.3 μm.

## 4. Conclusions

This manuscript is a comparative study on the SPIF process with the use of coated and uncoated tools led by an industrial robot.

The variation in the deformation forces for a part with a pyramidal trunk shape, the ANOVA analysis for experimental research on the maximum deformation force in the incremental forming process, numerical simulation with LS-DINA software, 3.2 version, of incremental deformation process, the variation in the accuracy of the incrementally formed part, and the quality of the surfaces (surface roughness) were all examined in this manuscript. The forces in incremental forming have components with rising values from the start of the procedure to the maximum value as a result of the hardening process. The F_x_ and F_y_ components oscillate between positive and negative values as a result of the change in the direction of the tool, and the F_z_ component oscillates with a certain amplitude and frequency due to the modification of the feed rate when changing the direction of the formation. Lower values of the forming components are ensured with the TiN-coated tool. Due to the kinematics of the forming process, deviations from the part in the drawing are observed, particularly in shape.

Based on the data obtained, the study limitations consist of the following:

- the temperature is not controlled in the deformed area (with the possibility of improving the material deformability);
- reduced precision of the process (need to use the counter-pouch (tool)/counter-matrice);
- the inclination angle of the wall cannot exceed 70° (under special conditions) because breaks and cracks occur;
- the process is limited and of a lower precision due to the use of a robot for traveling the tool, which has low rigidity. On the other hand, industrial robots can be used in the processing with incremental forming of areas of large assemblies that cannot be placed on the CNC milling machines’ table;
- long processing time compared to classic forming, which recommends it only for small and unique series production.

The practical recommendation that can be made is the use of tools coated with hard alloys (as titanium nitride) that, among others, improve the contact from the tool and the piece (deformed area). Also, it should be used counter-tool with a correlated movement with that of the tool (the kinematics of the equipment used, programming the movement of both tools and others).

## Figures and Tables

**Table 1:** Studies from the technical literature regarding the SPIF.

No. crt.	Material	Analysis	References
1	polymer sheets	optimization of forming forces	[68]
2	polycarbonate	formability limits (necking, fracture)	[69]
3	titanium and magnesium	surface roughness, design and production of parts, maximum forming angle	[70]
4	AZ31B-H24 (magnesium sheet)	temperature effect on roughness, microstructure and hardness, coating of parts using electrospun polycaprolactone	[71]
5	AA 1050 (aluminium alloy sheet)	role of heat in formability, comparation between elevated temperature incremental forming and conventional method	[72]
6	Ti-6Al-4V (titanium sheet)	analysis of forming force, surface roughness, geometric accuracy, thickness profile, micro-hardness for induction heating SPIF; finite element analysis—FEA (strain and stresses), Arrhenius model	[73]
7	Ti-6Al-4V (titanium sheet)	tensile test, stretch forming experiments (at 400 °C), FEA	[74]
8	Al alloy 5083 (aluminium alloy sheet)	surface roughness, deformation of thin-walled, mathematical models, optimization	[75]
9	Al2219-O, AA2219-T6 (aluminium alloy sheet)	tool rotational speed influence on tensile strength, microstructure analysis	[76]
10	AA 2014-T6 (aluminium alloy sheet)	surface roughness, Taguchi’s analysis, input parameters influence	[77]
11	AA1050-H111 (aluminium alloy sheet)	fracture forming limit, failure mechanism, ductile fracture, tensile stress, process parameter influence	[78]
12	AA 5052 (aluminium alloy sheet)	formability, optimization, variable wall angle, orthogonal array	[79]
13	AA1200 H14 (aluminium alloy sheet)	formability analysis, ANOVA analysis, microstructure	[80]
14	AA 1050 (aluminium alloy sheet)	residual stresses, hole drill test, Taguchi analysis	[81]
15	St12 (mild steel sheet)	surface roughness, Taguchi analysis, ANOVA methodology	[82]
16	AA1050-H14 (aluminium alloy sheet)	surface profile, influence of process parameters	[83]
17	Monolithic geometry	performance, strategies of experimental conditions, applications	[84]
18	AA1050 (aluminium alloy sheet)	microstructure, texture based analysis, structural and morphological analysis	[85]
19	AA2024, AA6061 (aluminium alloy sheet)	void coalescence and cluster, ductile fracture, GTN model, FEA	[86]
20	Al 6063-T6 (aluminium alloy sheet)	residual stress, nanoindentation fractal geometry	[87]
21	LITECOR^®^ composite material (metal-plastic)	residual stresses, structural and morphological analysis	[88]
22	AA1100 (aluminium alloy sheet)	hardness, artificial neural network, relative importance	[89]
23	TA1 (titanium sheet)	FEM, tensile test, thinning ratio, forming strain	[90]
24	titanium and titanium alloy sheets	microstructure, friction, lubrication	[91]
25	AL1060 (aluminium alloy sheet)	ultrasonic vibration, forming force, experimental verification, analytical modelling	[92]
26	2024-T3, 7075-T6 (aluminium alloy panels)	surface finish, surface roughness, surface topography, artificial neural networks modelling	[93]

**Table 2:** Experimental data with maximum values of force components.

No.	Tool	Feed Rate, Vs [mm/min]	Forming Step, Pi [mm]	F_z_ [kN]	F_x_ [kN]	F_y_ [kN]
1.	uncoated	5500	0.28	1.2	0.4	0.35
2.	uncoated	5500	0.56	1.39	0.38	0.4
3.	uncoated	8500	0.28	1.0	0.21	0.17
4.	uncoated	8500	0.56	1.41	0.4	0.47
5.	coated with TIN	5500	0.28	1.25	0.37	0.35
6.	coated with TIN	5500	0.56	1.38	0.42	0.44
7.	coated with TIN	8500	0.28	1.26	0.4	0.41
8.	coated with TIN	8500	0.56	1.41	0.37	0.41

**Table 3:** Numerical and experimental results for forces—case 1.

Case	Feed Rate	Forming Step	Maximum Force Fz	Maximum Force Fx	Maximum Force Fy
	[mm/min]	[mm]	[N]	[N]	[N]
Experimental	5500	0.56	1390	380	400
Simulation	5500	0.56	1395	410	420
Diference [%]			0.36	7.89	5

**Table 4:** Numerical and experimental results for forces—case 2.

Case	Feed Rate	Forming Step	Maximum Force Fz	Maximum Force Fx	Maximum Force, Fy
	[mm/min]	[mm]	[N]	[N]	[N]
Experimental	5500	0.56	1380	420	440
Simulation	5500	0.56	1490	440	450
Diference [%]			7.97	4.76	2.27

**Table 5:** Analysis of variance of factors on the maximum force component F_z_.

Factor	No. Degrees of Freedom (DF)	Adj. MS	F Value	Threshold of Significance, *p*	Statistical Significance *
Tool	1	0.0968	11.90	0.026	S
Feed rate, V_s_	1	0.005	0.61	0.477	NS
Forming step, P_i_	1	0.0072	0.88	0.400	NS

* S—statistically significant; NS—not statistically significant.

**Table 6:** Analysis of variance of factors on the maximum force component F_x_.

Factor	No. Degrees of Freedom (DF)	Adj. MS	F Value	Threshold of Significance, *p*	Statistical Significance *
Tool	1	0.004513	1.31	0.316	NS
Feed rate, Vs	1	0.010513	3.06	0.155	NS
Forming step, Pi	1	0.002113	0.61	0.477	NS

* S—statistically significant; NS—not statistically significant.

## Data Availability

Not applicable.
